# ‘Decision support system (DSS) for prevention of cardiovascular disease (CVD) among hypertensive (HTN) patients in Andhra Pradesh, India’ – a cluster randomised community intervention trial

**DOI:** 10.1186/1471-2458-12-393

**Published:** 2012-05-31

**Authors:** Raghupathy Anchala, Hira Pant, Dorairaj Prabhakaran, Oscar H Franco

**Affiliations:** 1 Department of Public Health & Primary Care, University of Cambridge, Strangeways Research Laboratory, Wort's Causeway, Cambridge, CB1 8RN, United Kingdom; 2 Public Health Foundation of India, Indian Institute of Public Health, Hyderabad, India; 3 Centre for Chronic Disease Control, New Delhi, India; 4 Public Health Foundation of India, New Delhi, India; 5 Department of Epidemiology, Erasmus MC, Rotterdam, The Netherlands

## Abstract

**Background:**

Very few studies having decision support systems as an intervention report on patient outcomes for cardiovascular disease in the Western world. The potential role of decision support system for the management of blood pressure among Indian hypertensives remains unclear. We propose a cluster randomised trial that aims to test the effectiveness and cost effectiveness of DSS among Indian hypertensive patients.

**Methods:**

The trial design is a cluster randomised community intervention trial, in which the participants would be adult male and female hypertensive patients, in the age group of 35 to 64 years, reporting to the Primary Health Care centres of Mahabubnagar district, Andhra Pradesh, India. The objective of the study is to test the effectiveness and compare the cost effectiveness and cost utility among hypertensive subjects randomized to receive either decision support system or a chart based algorithmic support system in urban and rural areas of a district in the state of Andhra Pradesh, India (baseline versus 12 months follow up). The primary outcome would be a comparison of the systolic blood pressure at 0 and 12 months among hypertensive patients randomized to receive the decision support system or the chart based algorithmic support system. Computer generated randomisation and an investigator and analyser blinded method would be followed. 1600 participants; 800 to each arm; each arm having eight clusters of hundred participants each have been recruited between 01 August 2011 - 01 March 2012. A twelve month follow up will be completed by March 2013 and results are expected by April 2013.

**Discussion:**

This cluster randomized community intervention trial on DSS will enable policy makers to find out the effectiveness, cost effectiveness and cost utility of decision support system for management of blood pressure among hypertensive patients in India. Most of the previous studies on decision support system have focused on physician performance, adherence and on preventive care reminders. The uniqueness of the proposed study lies in finding out the effectiveness of a decision support system on patient related outcomes.

**Trial registration:**

CTRI/2012/03/002476, Clinical Trial Registry - India.

## Background

Compared with all other countries, India suffers the highest loss in Potentially Productive Years of Life Lost (PPYLL), due to deaths from cardiovascular disease in people aged 35–64 years (9·2 million PPYLL in 2000, with a projected loss of 17.9 million PPYLL by 2030) [1]. CVD accounted for 29% of deaths and 11% of all Disability Adjusted Life Years (DALYs) in India (all ages, 2005) [2]. Hypertension is directly responsible for 57% of all stroke deaths and 24% of all coronary heart disease deaths in India [3]. The World Health Organization rates hypertension as one of the most important causes of premature death worldwide [4]. The global and regional burden of disease and risk factors study (2001), in a systematic analysis of population health data for attributable deaths and attributable disease burden, has ranked Hypertension in South Asia as second only to child underweight for age [5].

In an analysis of worldwide data for the global burden of hypertension, 20.6% of Indian males and 20.9% of Indian females were suffering from hypertension in 2005 [6]. The rates for hypertension in % are projected to go up to 22.9 and 23.6 for Indian males and females respectively by 2025 [6]. Recent studies from India have shown the prevalence of hypertension to be 25% in urban and 10% in rural subjects in India [3,7-9].

However, only about 25.6% of treated patients had their blood pressure under control, in a multi center study from India on awareness, treatment and adequacy of control of hypertension [10].

Decision support systems have been defined as the tools that help clinicians decide on a course of action in response to an understanding of the patient’s status. These have been devised to improve the management of hypertension and to help the Physician prescribe evidence based standardized medical care that will result in achieving adequate blood pressure control among hypertensive patients. DSS is a software that helps the Physician to (1) undertake a thorough evaluation of risk factors (2) to classify the risk level (3) to follow a software prompted algorithmic guideline based drug management (which has been developed based on Indian Hypertension guidelines II [11] (2007) and (4) to give alerts on the counseling on lifestyle changes and adherence to medication. Mixed results have been shown for DSS in the management of hypertension in the developed world for patient outcomes, but have shown that they improve the Physician performance [12-15]. An improvement in the quality of antihypertensive treatment, concurrently leading to a considerable reduction in drug costs have been shown for DSS [16].

Very few studies having Decision support systems (DSS) as an intervention report on patient outcomes for cardiovascular disease in the Western world. The potential role of DSS for the management of blood pressure among Indian hypertensives remains unclear. We propose a cluster randomised trial that aims to test the effectiveness and cost effectiveness of DSS among Indian hypertensive patients.

### Research question(s)

Are Decision Support Systems effective in lowering the blood pressure (therefore the risk for CVD) and improving the Quality of Life among hypertensive subjects in a Low and Middle Income setting? Are Decision Support Systems (DSS) cost effective in hypertensive subjects for prevention of cardiovascular diseases?

## Methods

### Objective

The objective of the study is to test the effectiveness and compare the cost effectiveness and cost utility among hypertensive subjects randomized to receive DSS or a chart based algorithmic support system in urban and rural areas of a district in the state of Andhra Pradesh, India (baseline versus 12 months follow up).

### Aim

To develop, pilot test and implement a decision support system for hypertensive subjects

1. To recruit and follow up hypertensive subjects for 12 months

2. To evaluate the effectiveness of DSS on CV risk reduction and quality of life scores

3. To compare the CEA and CUA among hypertensive subjects

#### Primary end point

· To compare the SBP (Systolic Blood Pressure) at 0 and 12 months among hypertensive patients randomized to receive the DSS or the chart based algorithmic support system

#### Secondary end point

· To compare the Quality of Life (QoL) scores at 0 and 12 months among hypertensive patients randomized to receive the DSS or the chart based algorithmic support system

· To compare the Cost effectiveness of the DSS versus the chart based algorithmic support system at the end of 12 months among hypertensive patients.

· To compare the Cost utility of the DSS versus the chart based algorithmic support system at the end of 12 months among hypertensive patients.

#### Inclusion criterion

1. Adult male and female Indians in the age group of 35 – 64 years

2. Systolic blood pressure (SBP) of 140 mm Hg or greater and/or diastolic blood pressure (DBP) of 90 mm Hg or greater (irrespective of antihypertensive medications)

3. Informed written Consent form

#### Exclusion criterion

Hypertensive subjects who have been hospitalized within the last 12 months and subjects with a history of cancer (physician certified).

**Figure 1:**
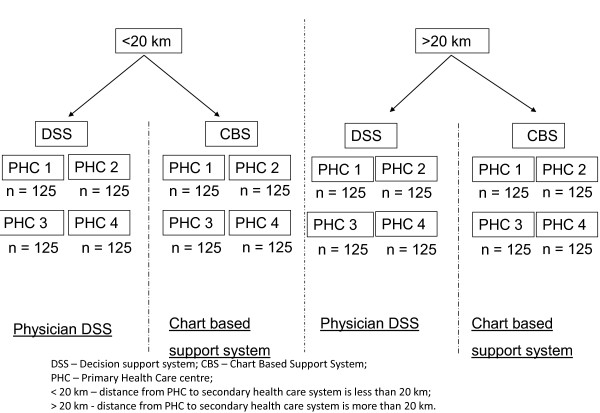
Plan for Randomisation.

Randomly chosen Primary Health Care centers (PHCs), matched on population size and literacy rate, from a district in the state of Andhra Pradesh, India have been cluster randomized to receive a DSS or a chart based algorithmic support system (Figure 1). The study site, Mahabubnagar district (AP) has four revenue divisions (Wanaparthy, Nagarkurnool, Gadwal and Narayanpet). Demographic details of all the PHCs, stratified by the revenue divisions in Mahabubnagar district, were line listed. The male to female ratio, age group distribution in the PHCs (based on census 2001 data) will be collected from the Andhra Pradesh State Health and Revenue departments. The PHCs will be stratified based on similarity in the study age group demography details (35-64 years of age) and gender distribution. Two PHCs, per intervention arm, will be randomly chosen from each of the four divisions.

Physicians from the PHCs, who have been randomised to receive the DSS arm would necessarily be trained and instructed to follow the algorithms and prompts which would arise out of the computer based software, for management of BP. DSS is a software that helps the Physician to (1) undertake a thorough evaluation of risk factors that hypertensive patients may have for developing a cardiovascular disease, (2) to classify the risk level, (3) to follow a software prompted algorithmic guideline based drug management (which would be developed based on Indian Hypertension guidelines II – 2007) and (4) to give alerts on the counselling on lifestyle changes and adherence to medication.

The chart based algorithmic support system will have the guidelines and lifestyle advices printed as a poster format which would then be pasted on the wall of the PHC clinics. The Physicians from the PHCs, who have been randomised to receive the chart based support arm, would be instructed to follow the poster based guidelines for management of BP. The risk factors that need to be specifically elicited, classification of risk among hypertensive subjects, flow chart for drug management and advice for lifestyle interventions would be included as a simple flow chart.

**Figure 2:**
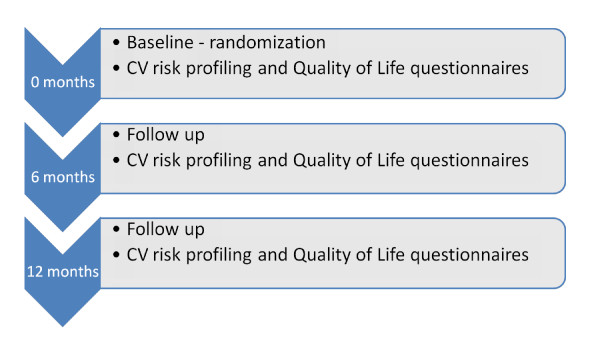
Follow up of patients.

The DSS will be developed, pilot tested in 10% of sample size and then implemented. Physician advice on lifestyle interventions, which would include counselling on reduction of daily salt intake (reduction in papad and pickle intake) and locally relevant fatty food items (ghee, vanaspati and dalda), advice on benefits of brisk walking for 30 min daily (5-7 days in a week), advice on benefits of smoking cessation and adherence to treatment would be available for patients randomized to DSS intervention arm at 0, 6 and 12 months and at 0 month for patients randomized to receive chart based algorithmic support system. Physical measurements such as height, weight, waist circumference, pulse and Blood Pressure (BP) would be measured by a digital BP monitor at 0, 6 and 12 months after study entry (Figure 2).

### Blood pressure and pulse measurement

All Blood Pressure (BP) measurements taken by the Physician will be performed in a standardised way using digital blood pressure equipments (Model M5, Omron) supplied and validated for the study. BP will be measured on the right upper arm in the sitting position, after a rest of 5 minutes. Using an appropriate sized cuff (which will be recorded) connected to a digital device, and the same arm (which will be recorded), at a similar time of day (which will be recorded), two measurements will be taken at a minute interval. Instructions will be given to the Physicians to ensure that the lower edge of the bladder be placed 2-3 cm above the position of maximal pulsation of the brachial artery in the arm, just above the antecubital fossa. Care would be taken to ensure that the cuff fits firmly, comfortably and is well secured. The mean of the two readings will be used for analysis.

Pulse measurements will be recorded by the digital blood pressure equipment (Model M5, Omron), after a rest of 5 minutes. The digital BP equipment records the systolic, diastolic BP and the pulse in the same sitting, which would be recorded. The average of the two readings will be used for the analysis.

### Height, weight and waist circumference measurements

The participants would be weighed in light indoor clothing with a digital weighing machine (Tanita weighing Scale) with 100 g accuracy, which would be standardized across all the centers. Height would be measured in bare feet with Seca 213 Portable Freestanding stadiometer, accurate to 1 mm. Care would be taken to ensure that all the PHCs have the same equipment make, type and validation procedures.

### Data collection

Data on known cardiovascular risk factors (age, sex, tobacco usage, history of diabetes mellitus, family history of premature coronary artery disease); any associated clinical conditions (physician certified cerebro vascular disease, cardiovascular disease, renal, vascular diseases); target organ damage (physician certified left ventricular hypertrophy, hypertensive retinopathy and microalbuminuria); and quality of life scores (based on WHO BREF questionnaire will be collected at 0, 6 and 12 months after randomization.

### Quality of life questionnaire

World Health Organization (WHO) QoL BREF questionnaires, which have been validated in Indian settings for chronic diseases, would be utilised in this study. WHOQOL was designed as an international cross-culturally comparable quality of life assessment instrument [17]. This 26-item questionnaire has been tested and validated in Hindi language [17], and tested in Indian patients suffering from chronic diseases [18-20]. The questionnaire would be translated into the local language (Telugu) and back translated by two independent translators. A third reviewer would compare the translated and back translated versions. The finalised questionnaire will be field tested in 10% of the proposed sample size for cultural appropriateness. Questions which are deemed to hurt the cultural sensitivity would be modified based on pilot test results. This modified questionnaire will then be applied to the study. Patients would be asked to read and answer the questions on their own. In case of linguistic or other difficulties, the questionnaire would be administered by interview.

### Cost effectiveness analysis

A comparison of costs in monetary units with outcomes in quantitative non-monetary units would done in the Cost Effectiveness Analysis (CEA) [21]. Resources required for intervention and values attached to those resources and effects of treatment (either benefit or harm) and values attached to those effects for the both the groups will be compared to determine the cost effectiveness ratios. Cost effectiveness ratios, that is the cost/outcome of different interventions, enable opportunity costs of each intervention to be compared.

#### Perspective

A third party payer perspective will be employed in this project, as this would enable the tangible costs to be directly compared between both the intervention arms.

#### Costs

The value of all goods, services, and other resources consumed in providing health care or dealing with side effects or other current and future consequences of health care comprise the direct costs. Direct health care and direct non health costs will be measured in this study. The physician services, hospital services, drugs, costs of the DSS software development, costs for charts (posters) etc. involved in delivery of health care would comprise the direct health care costs. Transportation to and from the site of care would comprise the indirect costs. Marginal costs analysis would be done in which how outcomes change with changes in costs (e.g., relative to a comparator), would be analysed. The time horizon for the costs involved would be for a duration of a year.

#### Outcomes

A reduction or worsening of systolic BP would be compared (in non monetary terms). The time horizon would be for a duration of one year.

#### Discounting

Thus, costs and outcomes will be *discounted* relative to their present value (e.g., at a rate of three - five percent per year) so as to account for the effect of time on the value of the outcomes and costs, as they may have less value in the future. Opportunity costs, which would be reflected by discounting, would also be calculated.

#### Sensitivity analysis

Since any estimation of costs and outcomes has an uncertainty element attached to it, a sensitivity analysis, which would include a change in discount rates, would be done to account for the changes in the cost-effectiveness planes of one intervention compared to another.

#### Cost Effectiveness Ratio (CER)

The costs and outcomes for both the outcomes will be calculated to yield a CER.

(1) $$\text{CER}=\{\left(\text{Cost of DSS}–\text{Cost of chart based}\right)/(\text{Primary outcome of DSS}–\text{primary outcome of chart based intervention})\}$$

### Cost utility analysis (CUA)

Measures costs in monetary units with outcomes in terms of their utility attribute [Quality Adjusted Life Years (QALYs)], i.e. cost per QALY. Cost utility would be measured by Incremental Cost Effectiveness Ratios (ICERs) between both the groups. ICER is a ratio between the differences in the cost involved between the intervention proposed (in Indian National Rupees) and the difference in QALYs between the groups.

Health Related Quality of Life (HRQoL) would be measured on a Visual Analogue Scale (VAS) preference scale with a maximum score of 100 (perfect or best imaginable health) and minimum score of 0 (a quality of life which equals worst health imagined) to measure the value of the health state. The weights for the quality will be based on the (1) preferences of the participants, (2) measured on an interval scale and (3) anchored on perfect health and death. The value of a health state would then be combined with the time spent to calculate the total QALYs. Usefulness, interpretability, and responsiveness were the determining factors to choose VAS as the preferred way to measure the utility of the present health state.

#### Sample size, sampling and recruitment

Table 1 gives the details of the sample size calculations. To detect a difference of 4 mm Hg Systolic Blood Pressure (SBP), the individual randomization sample size has been calculated at 239 subjects per intervention arm (SD of 19.5 mm for SBP [22]), with a 80% power and an alpha of 0.05. Since, cluster randomization is being attempted in this study, the individual randomization sample size has to be adjusted by a design effect of 2.98 [design effect = 1+ (size of cluster -1) * Intra cluster Correlation (ICC)]. The ICC for the clusters among the Mahabubnagar district, AP, India (study site) has been calculated to be 0.02 (based on the Indian sentinel surveillance study done on a representative sample from 10 sites in India). Hence, the cluster adjusted sample size is 713 hypertensive patients (8 clusters with 125 subjects in each cluster to allow for a 20% loss to follow up) per intervention arm to detect a 4 mm Hg difference in Systolic Blood Pressure (SBP) with a power of 80% and an alpha of 0.05. After adjusting for the coefficient of variation among the various clusters (CV = 0.25), the sample size per intervention arm is 741, i.e. a total of 8 clusters (with equal cluster size of 100 each) would be required per intervention arm.

**Table 1:** sample size calculations

**Detectable difference in SBP between both the groups**	**80% power with an ICC of 0.02**				**90% power with an ICC of 0.02**			
	**Sample size required for individual randomization per arm**	**Sample size required for cluster randomization per arm (DE = 2.98)**	**Sample size required for cluster randomization with CV = 0.25 (DE = 3.1)**	**Minimum no of clusters with a cluster size of 100**	**Sample size required for individual randomization per arm**	**Sample size required for cluster randomization per arm (DE = 2.98)**	**Sample size required for cluster randomization with CV = 0.25 (DE = 3.1)**	**Minimum no of clusters with a cluster size of 100**
4 mm of Hg	374	1115	1160	24	500	1490	1550	32
5 mm of Hg	**239**	**713**	**741**	**16**	320	954	992	20
6 mm of Hg	166	495	515	12	222	622	689	14

*ICC* = Intra Cluster correlation; *DE* = design effect; *CV* = coefficient of variation.

Randomly chosen 8 PHCs – 4 urban and 4 rural (homogenous in age and gender distribution) for each intervention arm from a district in the State of AP, India would form the study centers. Each PHC has an out patient load of 50-100 patients/day. 2- 4 hypertensive patients are being envisaged to be recruited per day by the Physician per PHC from the patients attending the out patient clinic in the PHC. 10- 20 patients per week per PHC, 20- 40 per month per PHC and 125 patients in 4-6 months. Study recruitment will last from June 2011 to Dec 2011.

#### Ethical principles

The participants would be given sufficient time to understand the contents of the PIS (containing the study purpose, duration, study procedures, right to withdrawal from the study, details of patient confidentiality, ethical committee clearances, the study team details), and encouraged to ask questions regarding any aspects of the study procedures that they have not understood. The written informed consent form will be read out aloud in the native language to the study participants. They will be given an opportunity to seek clarifications on any issues that have not been understood. The signatures would be obtained by the Physician who would be recruiting the hypertensive patients for the study. The Physician would also sign and date the informed consent form. One copy would be given to the patients and one copy would be retained at the study site.

## Discussion

Hypertension exerts a substantial burden on cardiovascular health status and health care systems in India. Treatment however can be costly and health care providers are interested in both whether treatments can offer improvements in disease burden and whether they represent value for money. Economic evaluations seek to resolve this issue by producing results that can be used to inform and assist the decision maker in allocating scarce health care resources. DSS have been in place in the developed world to achieve a balance between quality and cost of chronic disease health care. To the best of the author’s knowledge, there have been no randomized studies on DSS to enable policy makers to find out the effectiveness, cost effectiveness and cost utility analysis (CUA) of DSS for hypertensive patients in India. The cluster randomised community trial aims to test a decision support system among Indian hypertensive patients. Most of the previous studies on decision support system have focused on physician performance, adherence and on preventive care reminders. The uniqueness of the proposed study lies in finding out the effectiveness of a decision support system on patient related outcomes.

There are no known risks attributable to DSS per se. If the participant is receiving DSS, the software assisting the Physician prompts him or her to prescribe a particular class of drug to manage and control blood pressure. If the participant is receiving the chart based algorithmic support system, the decision to start or continue a drug is entirely up to the Physician’s judgement. The software will be developed and pilot tested and will be based on standard Indian Hypertension society guidelines (2007), which have been endorsed by the Association of Physicians of India and the Indian Cardiological Society. Adverse events that may arise due to the drugs will be monitored and any serious adverse events will be notified to the ethical committee within 24 hours. Should any serious adverse events arise the participants would be given the necessary care in the primary health care centre and if necessary will be transferred to the Rural Hospital. Substitution of the drugs causing adverse events is left to the decision of the Physician. Blood will be drawn by a qualified and an experienced phlebotomist, with due precautions. The quality of life questionnaire would be adapted from WHO BREF questionnaire which has been validated in India. Patient confidentiality will be maintained at data entry and data analysis stage.

## Conclusion

This cluster randomised community trial aims to test effectiveness, cost effectiveness and cost utility analysis of a decision support system among Indian hypertensive patients in comparison to a chart based algorithmic support system (that will have the guidelines and lifestyle advices printed as a poster format). The utility of DSS in management of hypertension will be tested in this pilot study. Subsequent scale up of DSS and future studies of DSS in management of different domains of cardiovascular diseases are envisaged based on the results of this pilot study.

## Ethical approval

Approved by the (1) Human Biology Research Ethics Committee, University of Cambridge, United Kingdom and (2) Institutional Ethics Committee, Public Health Foundation of India, New Delhi, India.

## Competing interests

There is no conflict of interest between the funder and the authors.

## Authors’ contributions

RA and OHF had the idea for the study. RA developed the study protocol, envisaged the study design and setting, wrote the methods for the paper, and developed the statistical analysis plan. RA and OHF wrote the manuscript. HP developed the data base for data collection and data entry. HP and OHF contributed to the initial revision of the manuscript. DP supervised the methods and contributed to the critical revision of the manuscript before publication. All authors read and approved the final manuscript.

## Pre-publication history

The pre-publication history for this paper can be accessed here:

http://www.biomedcentral.com/1471-2458/12/393/prepub

## Data sharing

No additional data available.
